# Association between Functional Severity and Amputation Type with Rehabilitation Outcomes in Patients with Lower Limb Amputation

**DOI:** 10.1155/2014/961798

**Published:** 2014-10-21

**Authors:** Amol M. Karmarkar, James E. Graham, Timothy A. Reistetter, Amit Kumar, Jacqueline M. Mix, Paulette Niewczyk, Carl V. Granger, Kenneth J. Ottenbacher

**Affiliations:** ^1^Division of Rehabilitation Sciences, University of Texas Medical Branch, 301 University Boulevard, Mail Route No. 1137, Galveston, TX 77555, USA; ^2^Occupational Therapy Department, University of Texas Medical Branch, Galveston, TX, USA; ^3^Uniform Data System for Medical Rehabilitation, A Division of UB Foundation Activities Inc. and Department of Rehabilitation Medicine, University at Buffalo, Buffalo, NY, USA

## Abstract

The purpose of this study was to determine independent influences of functional level and lower limb amputation type on inpatient rehabilitation outcomes. We conducted a secondary data analysis for patients with lower limb amputation who received inpatient medical rehabilitation (*N* = 26,501). The study outcomes included length of stay, discharge functional status, and community discharge. Predictors included the 3-level case mix group variable and a 4-category amputation variable. Age of the sample was 64.5 years (13.4) and 64% were male. More than 75% of patients had a dysvascular-related amputation. Patients with bilateral transfemoral amputations and higher functional severity experienced longest lengths of stay (average 13.7 days) and lowest functional rating at discharge (average 79.4). Likelihood of community discharge was significantly lower for those in more functionally severe patients but did not differ between amputation categories. Functional levels and amputation type are associated with rehabilitation outcomes in inpatient rehabilitation settings. Patients with transfemoral amputations and those in case mix group 1003 (admission motor score less than 36.25) generally experience poorer outcomes than those in other case mix groups. These relationships may be associated with other demographic and/or health factors, which should be explored in future research.

## 1. Introduction 

Comprehensive medical rehabilitation in inpatient rehabilitation facilities (IRFs) that involve several clinicians (e.g., physiatrists, rehabilitation nurses, physical therapist, occupational therapist, clinical psychologists, and social workers) is an effective component in the continuum of care for patients following amputation. Postacute inpatient rehabilitation is associated with reduced mortality, decreased chances of reamputation, improved functional independence, increased procurement of prosthetic devices, and increased probability for discharge to community settings [1–4]. The IRF setting is also reported to be a cost-effective option for caring for patients following dysvascular amputations relative to skilled nursing facilities [3]. However, there is limited evidence regarding the overall effectiveness and/or efficiency of inpatient rehabilitation for patients with amputation [2, 4]. Prior research suggests that inpatient rehabilitation is underutilized by patients receiving dysvascular-related amputations; less than 10% of patients are admitted to an IRF, with the majority being discharged home or to a skilled nursing facility [5].

Amputation is one of the 13 eligible medical conditions for IRFs to meet the current “60%-rule” criteria (which mandates that 60% of patients admitting to IRF to have one or more of the 13 eligible medical conditions) under the IRF prospective payment system developed by the Centers for Medicare and Medicaid Services [6]. The basis for reimbursement under the IRF prospective payment system is a patient's impairment-specific case mix group (CMG). CMGs were developed to account for resource utilization requirements of patients with similar functional deficits and rehabilitation needs [6] and are often used as a proxy for patient functional severity. Amputation-related CMGs are derived from admission motor functional independence measure (FIM) instrument ratings within the inpatient rehabilitation facility patient assessment instrument [6]. There are three CMGs for amputation (CMG 1001 (FIM admission motor score greater than 47.65), CMG 1002 (FIM admission motor score greater than 36.25 and less than 47.65), and CMG 1003 (FIM admission motor score less than 36.25)). Neither the number (unilateral versus bilateral) nor the level (transtibial versus transfemoral) of the amputation(s) is directly factored into the prospective payment equation (CMG calculations). There is a lack of published information regarding the potential benefits of including definitive amputation characteristics along with CMG in models designed to predict the rehabilitation experiences and outcomes of patients following amputation [5].

The purpose of this study was to determine the impact of the type of lower limb amputation and functional level (CMGs) at admission on rehabilitation outcomes in patients receiving inpatient medical rehabilitation in a nationally representative sample of IRF patients in the United States. The primary objective of this study was to test utility of clinical characteristics (amputation levels) that can be observed by clinicians and case mix groups that are defined by the payers, which classify patients based on the admission motor scores and prospectively allocate resources (utilization of services and length of stay) at inpatient rehabilitation facilities. These adjustments might be helpful in refining case mix and prospective payment system for patients with lower limb amputation seeking inpatient rehabilitative services.

## 2. Methods

### 2.1. Study Design

The study was a secondary analysis of medical records from 901 IRFs that subscribe to the Uniform Data System for Medical Rehabilitation (UDS_MR_). The UDS_MR_ database is the world's largest nongovernmental registry for IRF data and accounts for over 70% of the market share in the United States. For this study, we extracted data related to patient demographics, health characteristics, and rehabilitation. The study was approved by the institutional review board (IRB) at primary author's institution.

### 2.2. Study Sample

Patients aged 18 years and older who received inpatient medical rehabilitation for lower limb amputation from October 2005 to December 2007 were included in our study sample.

The total eligible sample using amputation rehabilitation impairment group codes (05.3, 05.4, 05.5, 05.6, and 05.7) was 102,049 cases. We included only those cases admitted for initial rehabilitation, admitted directly from acute hospitals, those without rehabilitation program interruption, and those living in the community prior to their acute admissions.

We excluded cases with missing information on the type of lower limb amputation and those died during rehabilitation stay. The final sample contained 26,501 patients with lower limb amputations.

### 2.3. Independent Variables

Lower limb amputation category was assigned according to four impairment codes for lower limb amputation: unilateral transtibial (05.4), unilateral transfemoral (05.3), bilateral transtibial (05.7), and bilateral transfemoral (05.5). Transtibial amputation of one side and transfemoral amputation of the other side (05.6) were included in the bilateral transfemoral group. Such classification was made, as the clinical characteristics of cases with transfemoral and transtibial amputation were similar to that of bilateral transfemoral group compared to bilateral transtibial group.

Case mix groups (CMGs) are used to group patients with similar clinical characteristics in order to estimate resources that will be utilized in the IRF. The basis for calculating CMGs in lower limb amputation patients is from weighted motor FIM ratings calculated at admission. The weighted motor FIM rating methodology was created by CMS as a way of accounting for the impact of each FIM motor item on the cost of providing care in the IRF. Patients with lower limb amputation are categorized into three CMGs: CMG 1001 (FIM motor greater than 47.65), CMG 1002 (FIM motor greater than 36.25 and less than 47.65), and CMG 1003 (FIM motor less than 36.25).

### 2.4. Rehabilitation Outcome Variables

Length of stay is the total number of days spent in IRF. For patients who were transferred to an acute-care setting and returned to IRF within three days, the days spent in acute-care were not included in computing the length of stay variable. Functional status was assessed by the FIM instrument items within the inpatient rehabilitation facility patient assessment instrument. The functional items of the inpatient rehabilitation facility patient assessment instrument are administered within three days of admission to IRF and again within three days of discharge [7]. The FIM instrument includes a total of 18 items which span two domains (motor and cognitive) and six subdomains (self-care, sphincter control, transfers, mobility, communication, and social integration). Ratings for each item range from 1 (total assistance) to 7 (complete independence). Total FIM ratings are derived by summing all 18 individual items to come up with a composite score which ranges from 18 to 126 [8]. The FIM instrument has been demonstrated to be a valid and reliable measure of functional status in a variety of IRF patient populations [9].

Discharge setting was dichotomized as community discharge (home, board and care, and transitional or assisted living) versus not community discharge (intermediate care, skilled nursing facility, acute unit own facility, acute unit another facility, chronic hospital, rehabilitation facility, alternate level of care, subacute setting, and others).

### 2.5. Covariates

Demographic factors included age, gender, race/ethnicity (non-Hispanic white, black, Hispanic, and others), and marital status (married versus unmarried). Health characteristics included etiology for amputation (dysvascular versus nondysvascular, trauma-related, cancer-related, or any other etiology), which was computed using International Classification of Disease, Clinical Modification codes (ICD9-CM) associated with amputation [1], diabetes status (yes versus no, also identified through ICD9-CM codes), and a summed score for the total number of other nondiabetes comorbidities (range: 0–10).

### 2.6. Data Analyses

Patient demographic characteristics, health characteristics, and outcomes were stratified by amputation type. Univariate analyses were used to test for differences between amputation categories, using one-way ANOVA for continuous variables and chi-square tests for categorical variables. This screening was done in order to select the variables that are associated with the proposed outcomes of the study. Covariates with a significant association with the outcomes were included in the regression models. Two multiple linear regression models were constructed to determine the impact of independent variables (CMG and amputation level) on predicting rehabilitation outcomes (LOS and discharge functional rating) while controlling for other demographic and health characteristics (covariates). Similarly, a logistic model was constructed to determine the association between CMG and amputation type on likelihood for community discharge. We also tested for interaction between CMGs and amputation levels in the model. The six interaction terms controlled for in all regression models were CMG 1002 by bilateral transtibial, CMG 1002 by unilateral transfemoral, CMG 1002 by unilateral transtibial, CMG 1001 by bilateral transtibial, CMG 1001 by unilateral transfemoral, and CMG 1002 by unilateral transtibial. All statistical analyses were computed using PASW v18.0 (SPSS IBM) software.

## 3. Results

A total of 26,501 records of patients with lower limb amputation were identified. The mean age of the sample was 64.5 years (sd = 13.4), and nearly two-thirds (64%) were male. Unilateral transtibial amputation was the single largest amputation category: approximately 60% of the total sample. Approximately 80% of both bilateral and unilateral transtibial amputations were dysvascular-related. Diabetes is more frequently reported in patients with bilateral (77%) and unilateral (73%) transtibial amputations.  Table 1 shows patient characteristics and rehabilitation outcomes for the entire sample and is stratified by amputation category.

**Figure 1:**
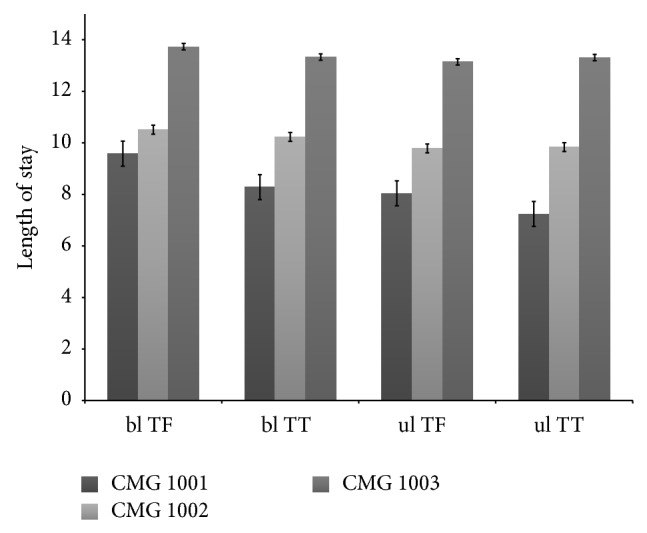
Length of stay by amputation levels for case mix group after adjustment of function. ul = unilateral, bl = bilateral, TF = transfemoral, and TT = transtibial.

**Figure 2:**
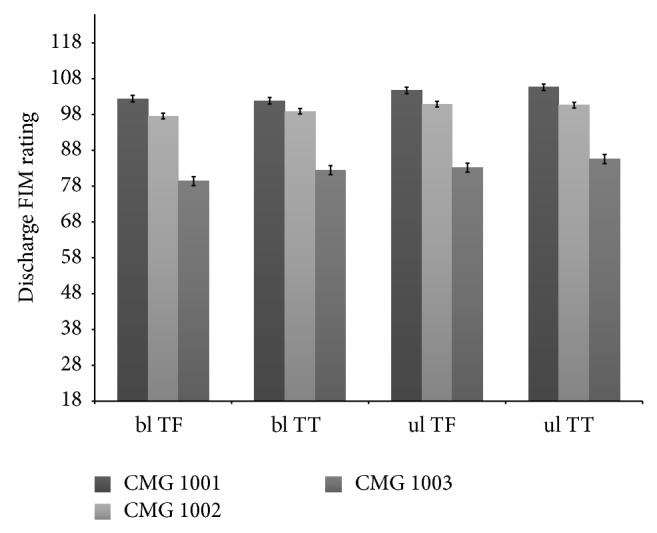
Discharge FIM rating by amputation levels for case mix group after adjustment of function. ul = unilateral, bl = bilateral, TF = transfemoral, and TT = transtibial.

**Figure 3:**
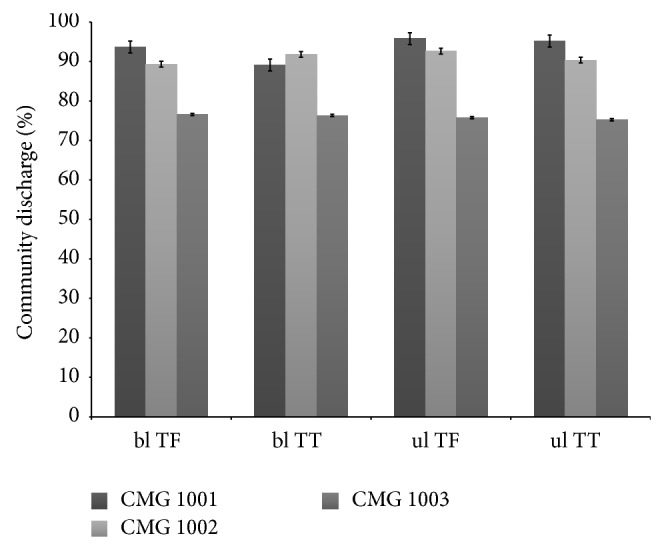
Community discharge by amputation levels for CMG after adjustment of function. ul = unilateral, bl = bilateral, TF = transfemoral, and TT = transtibial.

Without adjusting for other covariates, patients with bilateral transfemoral level of amputation under CMG 1003 had the highest LOS (13.7 days) as compared to patients with other levels of amputation and CMG. Those with unilateral transtibial level of amputation under CMG 1001 had the lowest LOS in IRF (7.2 days) (Figure 1). Discharge functional rating was lowest for those with bilateral transfemoral level of amputation under CMG 1003 (79.4) and highest for those with unilateral transtibial level of amputation under CMG 1001 (105.6) (Figure 2). Proportion of discharge to community was lowest among patients categorized into CMG 1003 compared to those under either 1002 or 1001, irrespective of amputation levels. This proportion was also lower for those patients under CMG 1002 than CMG 1001 for all except bilateral transtibial amputation level (Figure 3).

Tables 2 and 3 show the results of the linear and logistic regression analysis. CMG was strongly associated with all three outcomes; significant differences were observed between each CMG level. As expected, patients in CMG 1003 demonstrated the longest lengths of stay and lowest functional ratings at discharge and were least likely to be discharged home, as compared to both CMG 1001 and CMG 1002. Amputation category was also associated with all three outcomes. Patients with bilateral transfemoral amputations demonstrated the longest lengths of stay and lowest functional ratings at discharge. However, there was no significant association between amputation level and discharge to home in our sample. For LOS we found interaction between CMG 1001 and unilateral transtibial amputation category (*P* < .05). For discharge functional rating an interaction was significant for CMG 1002 and unilateral transtibial level of amputation (*P* < .05). For discharge to home interaction was only significant between CMG 1002 and unilateral transfemoral amputation level (*P* < .05) (Figure 3).

## 4. Discussion

Our study investigated the impact of case mix group and lower limb amputation type on inpatient rehabilitation outcomes. We analyzed data from 901 inpatient rehabilitation facilities in USA for the years 2005–2007.  More than 75% of the patients had amputation due to dysvascular and/or peripheral vascular disorder conditions. The prevalence of diabetes alone was 66%. Presence of diabetes associated with foot ulcers or other dysvascular conditions is a common reason for lower limb amputation in patients over the age of 40 [3, 5, 10, 11]. Lifetime risk of lower limb amputation among patients with diabetes is approximately 15% [12]. As the incidence rate of dysvascular conditions continues to increase, so will the number of lower limb amputations. Older adults are significantly more likely to experience dysvascular-related amputations compared to younger patients, who experience relatively more trauma-related amputations [13]. Higher mortality, increased numbers of reamputation, and greater cost are associated with dysvascular-related amputations compared with other causes (e.g., trauma or cancer) [2, 3].

In our study, patients who were assigned to lower level CMGs at admission showed better outcomes related to LOS and discharge functional rating, as compared to those assigned to higher level CMGs. These findings are of no surprise, as CMGs are used to estimate use of resources by patients, and higher CMG categories are expected to have higher utilization compared to patients assigned lower CMGs. In addition, we also found that patients with bilateral transfemoral amputations were more likely to stay longer in IRFs compared to those with unilateral amputations. Others have shown higher utilization in terms of longer lengths of stay and increased costs associated with higher levels of amputation (e.g., transfemoral), compared to lower levels of amputation (e.g., transtibial or foot) [2, 3]. Our results of a positive association between age and LOS also support previous findings demonstrating the increased risk of amputation with age resulting in higher resources use. We also found a significant association between presence of diabetes and longer LOS in our sample.

Regarding discharge functional rating, patients with higher level bilateral amputations had significantly lower FIM discharge ratings compared to those with lower level and/or unilateral amputations. A study by Pezzin and colleagues reported lower physical rating (on SF-36) associated with higher amputation levels for their sample from a trauma center [14]. Findings from the current study are important as FIM items are a core component of functional assessment in IRF, and discharge FIM ratings are associated with long-term recovery in various impairment categories [15]. In our study, age was negatively associated with discharge FIM rating, which supports previous conclusions regarding poor outcomes associated with higher age in patients with lower limb amputation [14]. Additionally, we found that diabetes was negatively associated with discharge FIM rating.

Higher CMG assignment (1003) was also associated with lower probabilities of discharge to the community compared to patients assigned to lower CMGs (1001 and 1002). However, we did not find an association between amputation level and community discharge in our sample. Patients with unilateral transtibial, unilateral transfemoral, and bilateral transtibial had higher likelihood of being discharged to community settings compared to those with bilateral transfemoral amputations. Age did not have a significant impact on discharge destination in our sample. Among other covariates, being married versus unmarried was strongly associated with community discharge. This finding is consistent with previous reports showing a strong relationship between the availability of caregiving support and likelihood of home (community) discharge for patients with stroke [16].

There are limitations to consider in the current study. First, we did not include health insurance status in the regression models. Availability and type of health insurance could have a direct impact on rehabilitation processes (access and utilization of services) and certain outcomes such as length of rehabilitation stay and discharge setting (community versus other settings). Secondly, the data were limited to the years following substantial changes in the way CMGs for amputation being determined. In October 2005, the Centers for Medicare and Medicaid Services introduced the weighted motor index and reduced the number of amputation-related CMGs from five to three. Future research is needed to examine the impact which changes in CMG classification for amputation had on rehabilitation outcomes. This study did not use a standardized comorbidity index; instead we summed all comorbidities and presented them as numbers. This was done in order to separate all diabetes comorbidities from others. Also, CMGs were developed by the Centers for Medicare and Medicaid Services as a way to allocate resources prospectively for rehabilitation impairment categories. Therefore we could only assume that the prospective resources allocation would also be pertinent to patients in our sample under the age of 65 and those who were non-Medicare enrollees. It is also important to note that we did not look at date of functional assessment, which could have been later than admission dates and earlier than discharge date. A key strength of this study is that it includes a large cohort of patients with lower limb amputation undergoing inpatient medical rehabilitation. Other strengths include use of case mix groups, which is an understudied area of research in medical rehabilitation.

## 5. Conclusion

Both CMG and amputation categories were significant predictors of rehabilitation outcomes: rehabilitation length of stay and discharge functional rating. CMG and amputation levels were associated with discharge to home, along with being married. Clinicians (e.g., physiatrist, rehabilitation nurses, physical therapist, and occupational therapist) are involved in provision of rehabilitation services for individuals with lower limb amputation at inpatient rehabilitation settings. This investigation indicates that use of amputation categories is important in projecting outcomes associated with provision of rehabilitation services for patients with lower limb amputation. Including something as easily observable as amputation category along with the standard CMG level may help clinicians, researchers, and policymakers better understand and predict the unique rehabilitation needs and experiences of different patients with lower limb amputations.

## Figures and Tables

**Table 1:** Demographic characteristics and rehabilitation outcomes by amputation levels.

	Total	ul TT	ul TF	bl TT	bl TF
*N*	26,501	15,798	7,495	1,610	1,598
Demographics					
Age∗	64.5 ± 13.4	63.8 ± 13.2	66.3 ± 13.6	62.6 ± 13.5	64.2 ± 13.1
Men (%)∗	64.0	65.7	59.9	68.5	62.5
Race/ethnicity∗%					
Non-Hispanic white	63.5	63	66.7	57.5	58.3
Black	21.0	20.4	20.2	25.8	26
Hispanic	7.6	8.5	6.2	7.1	6.7
Other	7.9	8.1	6.9	9.6	8.9
Married (%)∗	50.4	50.7	50.1	52.9	47.6
Etiology-dysvascular (%)∗	76.5	79.8	68.7	80.4	76.8
Diabetes (%)∗	66.0	72.8	50.3	76.6	62.1
Comorbidities (sum)∗	2.2 ± 1.4	2.3 ± 1.4	1.9 ± 1.3	2.7 ± 1.5	2.3 ± 1.5
Case mix group∗(%)					
CMG1001	8.3	8.5	8.4	8.1	6.7
CMG1002	29.3	31.6	27.1	24.8	21.7
CMG1003	62.3	59.9	64.5	67.1	71.6
Admission functional rating					
FIM motor admission∗	38.8 ± 11.4	39.6 ± 11.1	37.9 ± 11.8	37.6 ± 11.6	35.5 ± 11.8
FIM cog admission∗	27.3 ± 6.4	27.5 ± 6.2	26.9 ± 6.6	26.9 ± 6.3	26.7 ± 6.7
FIM total admission∗	67.9 ± 16.4	69 ± 15.9	66.7 ± 17	66.2 ± 16.5	63.8 ± 17
Rehabilitation outcomes					
FIM motor discharge∗	55 ± 14.1	56.1 ± 13.5	54.2 ± 14.7	53.2 ± 14.1	50.4 ± 15.2
FIM cog discharge∗	29.5 ± 5.7	29.9 ± 5.5	29.2 ± 6	29.3 ± 5.8	28.6 ± 6.4
FIM total discharge∗	88 ± 19.4	89.5 ± 18.6	86.8 ± 20.2	85.7 ± 19.4	82 ± 21.2
Length of stay (days)∗	13.3 ± 6.5	13.2 ± 6.4	13.2 ± 6.5	13.6 ± 6.9	14.2 ± 6.8
Discharged home (%)	72.4	72.6	72.4	71.8	70.7

ul = unilateral, bl = bilateral, TF = transfemoral, and TT = transtibial.

∗A significant relationship between amputation category and denoted variable at *P* < .05.

Case mix groups (CMGs) are calculated from weighted admission FIM motor ratings: CMG 1001 (FIM motor greater than 47.65), CMG 1002 (FIM motor greater than 36.25 and less than 47.65), and CMG 1003 (FIM motor less than 36.25).

**Table 2:** Coefficient estimates from multiple linear regression models: predictors for length of stay and discharge FIM rating, source UDSMR database.

Variables	Length of stay		Discharge FIM rating	
	Coefficient estimate	Confidence interval (95%)	Coefficient estimate	Confidence interval (95%)
Age, yrs	0.03∗	0.02 to 0.032	−0.29∗	−0.31 to −0.27
Male	0.27∗	0.12 to 0.432	−1.27∗	−1.69 to −0.85
White	−0.24∗	−0.40 to −0.088	2.32∗	1.90 to 2.73
Married	−0.78∗	−0.93 to −0.63	−0.22	−0.62 to 0.18
Dysvascular	0.11	−0.06 to 0.29	−1.14∗	−1.61 to −0.66
Diabetes	0.35∗	0.18 to 0.52	−0.014	−0.45 to 0.43
Comorbid, sum	0.26∗	0.22 to 0.29	−0.53∗	−0.62 to −0.45
CMG 1003 (Ref)				
CMG 1002	−3.22∗	−3.96 to −2.5	18.17∗	16.23 to 20.11
CMG 1001	−4.16∗	−5.37 to −2.9	23.02∗	19.79 to 26.25
bl TF (Ref)				
bl TT	−0.41	−0.91 to 0.10	3.08∗	1.74 to 4.42
ul TF	−0.59∗	−0.98 to −0.19	3.76∗	2.72 to 4.81
ul TT	−0.42∗	−0.79 to −0.04	6.17∗	5.18 to 7.16
CMG 1002 × bl TT	0.13	−0.88 to 1.1	−1.76	−4.44 to .92
CMG 1002 × ul TF	−0.14	−0.93 to 0.66	−0.45	−2.56 to 1.66
CMG 1002 × ul TT	−0.25	−1.01 to 0.51	−3.15∗	−5.16 to −1.13
CMG 1001 × bl TT	−0.89	−2.53 to 0.76	−3.65	−8.01 to 0.70
CMG 1001 × ul TF	−0.95	−2.27 to 0.36	−1.46	−4.94 to 2.03
CMG 1001 × ul TT	−1.92∗	−3.18 to −0.65	−2.97	−6.32 to 0.38

ul = unilateral, bl = bilateral, TF = transfemoral, and TT = transtibial.

∗Significance at *P* < .05.

× = interaction term.

**Table 3:** Odds ratios from multiple logistic regression model.

Variables	Community discharge	
	Odds ratio	Confidence interval (95%)
Age, yrs	0.98	0.98 to 0.99
Male	1.13	1.06 to 1.20
White	0.97	0.91 to 1.03
Married	1.51∗	1.42 to 1.60
Dysvascular	0.94	0.88 to 1.01
Diabetes	1.06	0.99 to 1.13
Comorbid, sum	0.95	0.93 to 0.96
CMG 1003 (Ref)		
CMG 1002	2.56∗	1.86 to 3.52
CMG 1001	4.54∗	2.26 to 9.12
bl TF (Ref)		
bl TT	0.99	0.82 to 1.18
ul TF	0.96	0.83 to 1.10
ul TT	0.93	0.82 to 1.06
CMG 1002 × bl TT	1.36	0.86 to 1.68
CMG 1002 × ul TF	1.56∗	1.10 to 1.33
CMG 1002 × ul TT	1.20	0.86 to 1.67
CMG 1001 × bl TT	0.56	0.23 to 1.33
CMG 1001 × ul TF	1.60	0.73 to 3.47
CMG 1001 × ul TT	1.43	0.69 to 2.97

ul = unilateral, bl = bilateral, TF = transfemoral, and TT = transtibial.

∗Significance at *P* < .05.

× = interaction term.
